# Peroxynitrite and 4-Hydroxynonenal Inactivate Breast Cancer Resistance Protein/ABCG2

**DOI:** 10.1155/2019/3891535

**Published:** 2019-01-21

**Authors:** Svetlana Verenich, Phillip M. Gerk

**Affiliations:** Department of Pharmaceutics, School of Pharmacy, Virginia Commonwealth University, P.O. Box 980533, Richmond, VA 23298-0533, USA

## Abstract

Oxidative stress may arise from a variety of pathologies and results in the formation of toxic and reactive chemical species. Extensive research has been done to establish mechanisms of formation and cytotoxic effects of a number of different products of oxidation stress including peroxynitrite (PN) and 4-hydroxynonenal (4HNE). However, relatively few studies have investigated their effects on ATP-binding cassette (ABC) transporters. The objective of this investigation was to determine the effects of PN and 4HNE on BCRP/ABCG2. To eliminate the effect of metabolic enzymes, the experiments were carried out with inside-out* Sf9* membrane vesicles overexpressing BCRP/ABCG2 using riboflavin as a substrate. The experiments revealed that PN produced IC_50_ of about 31.2 ± 2.7 *μ*M, based upon initial concentrations. The IC_50_ for 4HNE was estimated to be 92 ± 1.4 *μ*M. Preincubation of membrane vesicles with either PN or 4HNE caused the maximal rate of transport (V_max_) to drop drastically, up to 19 times, with no or much smaller effect on K_m_. Thus, PN and 4NE can inhibit BCRP transport activity.

## 1. Introduction

Oxidative stress is a frequent complication of various disease conditions such as Alzheimer and Parkinson's diseases [1, 2], atherosclerosis [3], HIV [4], preeclampsia [5], rheumatoid arthritis [6], and diabetes [7] including gestational diabetes [8]. Different molecules associated with oxidative stress are involved in the development of pathogenesis; however peroxynitrite (PN) and 4-hydroxynonenal (4HNE) are the most studied. The former is a reaction product of superoxide and nitric oxide, while 4HNE is a product of lipid peroxidation of polyunsaturated fatty acids [9]. Both of them are implicated in damage, adduction, or inhibition of synthesis of DNA or RNA, cell apoptosis or necrosis, mitochondrial dysfunction, and lipid peroxidation [10, 11]. Typically 4HNE actively forms adducts with thiol-containing amino acids or substances such as glutathione and cysteine and with lysine, histidine residues of protein; PN, on the other hand, reacts with methionine and cysteine, as well as residues of tyrosine and tryptophan by oxidizing or nitrating them [9, 12]. All of the above indicate that PN and 4HNE are capable of damaging or disrupting the functionality of proteins. Thus, it was reported that PN could inhibit Na-K-, Ca^2+^-, and Mg^2+^-ATPases [13, 14]. However, the effect of PN and 4HNE on ATP-binding cassette (ABC) transporters has not been thoroughly investigated.

ABC transporters modulate the movements of nutrient or xenobiotics across the membrane by utilizing the energy from ATP. P-glycoprotein (ABCB1) and BCRP (breast cancer resistance protein) [15] are an important determinant of drug absorption and tissue penetration. So far, only Soszyński and Bartosz [16] have reported that peroxynitrite showed an inhibition of transport of S-conjugates modulated by ABCC1 (MRP1).

The purpose of this study was to demonstrate if 4HNE and PN can inhibit the transport of riboflavin mediated by BCRP/ABCG2 and identify half maximal inhibitory concentration (IC_50_) for both PN and 4HNE. To eliminate the interference of metabolism, the experiments were performed with* Sf9 *membrane vesicles overexpressing BCRP. Thus, this investigation provides evidence that both 4HNE and PN inactivate BCRP, with peroxynitrite being more potent.

## 2. Materials and Methods

### 2.1. Materials

Riboflavin was obtained from MP Biomedicals, Inc. (Solon, OH). Peroxynitrite (PN) and 4-hydroxynonenal (4HNE) were purchased from Cayman Chemical (Ann Arbor, MI) and stored at –80°C. The concentration of PN was determined by diluting in ice-cold 0.3N NaOH, determining the absorbance at 302nm and calculating the concentration based on the extinction coefficient for peroxynitrite (1670 M^−1^cm^−1^). All other reagents and solvents were obtained from Sigma-Aldrich (St. Louis, MO).

### 2.2. Sf9 Membrane Vesicle Preparation and Transport Study

Inside-out* Sf9* membrane vesicles overexpressing human BCRP or empty vector (i.e., lacking ABC transporters) were prepared as previously described [17, 18]. Briefly, a suspension of* Sf9 *cells was infected with titered viral stocks of BCRP or empty vector (EV) at a multiplicity of infection of 4. After 48 hours,* Sf9* cells were harvested, lysed, and homogenized; then their membranes were isolated by fractionation over 38% sucrose. Isolated membranes were vesiculated, snap frozen in liquid nitrogen, and stored at – 80°C. The membrane protein concentrations were determined by the modified Lowry protein assay with bovine serum albumin used as a standard. [19]

Transport experiments were carried out as described in Gerk et al. [20] with small modifications in the procedure. The final buffer concentrations were tris-HCL 10mM (pH 7.4), sucrose 250mM, 5 mM ATP or AMP, 10 mM phosphocreatine, 100 *μ*g/ml creatine phosphokinase, 10 mM MgCl_2_, riboflavin in deionized water, or dimethyl sulfoxide (0.5%) with or without reduced glutathione (1mM). For osmotic sensitivity experiments, sucrose concentrations from 250mM to 1M were used. For experiments with PN, tris-HCl was replaced with triethanolamine HCl 10mM (pH 7.4). ATP-dependent transport of riboflavin was conducted in 96-well PCR plates with a reaction volume of 60 *μ*l incubated at 37°C between 2 and 60 min. Transport activity was stopped by transferring 50 *μ*l to a 96-well filter plate with prewetted 0.45 *μ*m PVDF filters (Millipore Corporation, Bedford, MA) and by quick washing under vacuum with ice-cold stop buffer (3 x 200 *μ*l) to harvest membrane vesicles. To elute riboflavin, 100 *μ*l of water/dimethyl sulfoxide mixture (3:1) was added to the filter plate and centrifuged at room temperature for 8 min at 350 rcf_max_. The filtrate was collected in a black-sided clear bottom 96-well plate (Costar 3631, Corning Inc., Corning, NY). Riboflavin was quantitated using a microplate reader Synergy™ 2 (BioTek Instruments, Inc., Winooski, VT) with excitation and emission wavelengths of 450 and 528 nm, respectively. The ATP-dependent transport of riboflavin was estimated as a difference between the values obtained in the presence of ATP or AMP. For the experiments with PN and 4HNE, the membrane vesicles were preincubated with PN or 4HNE at 37°C for 30 min prior to the transport experiments.

### 2.3. Data Analysis for Transport Kinetics

Kinetic parameters were estimated from curve fitting using GraphPad Prism for Windows (version 5, GraphPad Software Inc., San Diego, CA) by means of unweighted nonlinear regression using the Hill equation and the sigmoidal dose-response equation.

All data values were expressed as a mean of 3 or 4 independent determinations. The effect of PN and 4HNE on ATP-dependent transport of riboflavin was compared by analysis of variance (one-way ANOVA) followed by Dunnett's posttest or unpaired* t*-test. A value of* p* < 0.05 (2-sided) was considered statistically significant. Goodness of fit of different models was compared using Akaike information criterion (AIC).

## 3. Results

**Figure 1:**
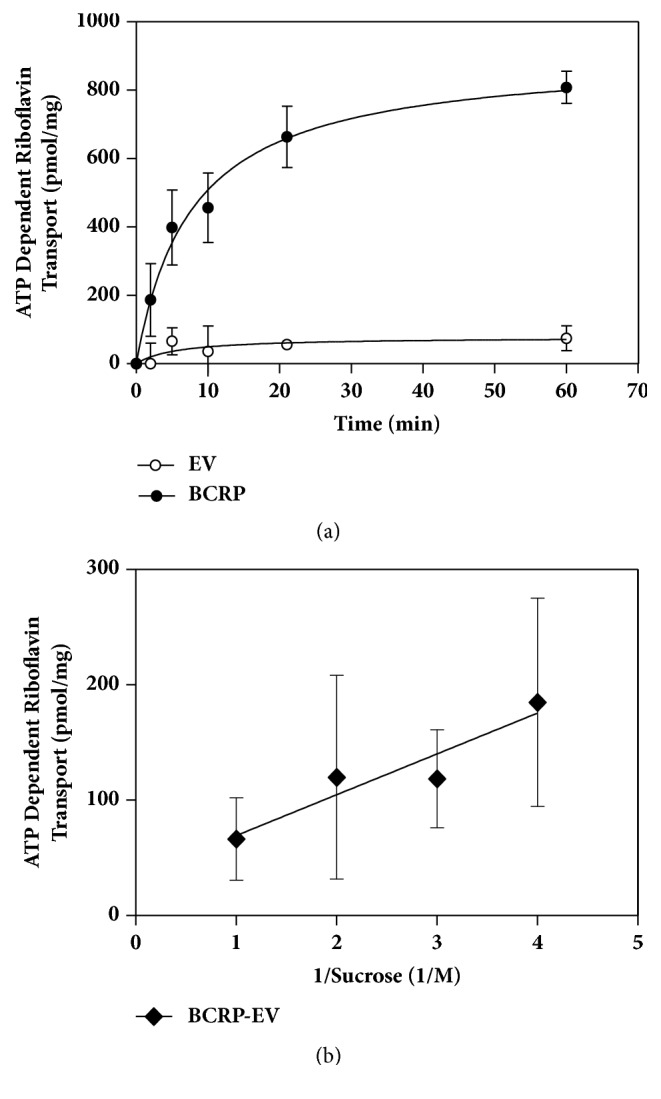
BCRP-mediated uptake of riboflavin into* Sf9* inside-out membrane vesicles. Each data point represents mean ± SD from 3-4 determinations, expressed as pmol riboflavin/mg protein. (a) Time course of riboflavin (10 *μ*M) uptake into* Sf9* membrane vesicles overexpressing BCRP/ABCG2 (filled circles) and EV vesicles (open circles). Protein amount is 10 *μ*g; (b) effect of sucrose (osmolarity) on ATP-dependent uptake of riboflavin (10 *μ*M) into* Sf9 *vesicles. The transport was determined after 5 min in the presence of varying sucrose concentrations (250-1000*μ*M).

In this work we investigated ATP-dependent uptake of riboflavin (vitamin B_2_) into inside-out* Sf9* membrane vesicles overexpressing BCRP/ABCG2. Thus, Figure 1(a) depicts that, in the presence of ATP, the membrane vesicles showed a time-dependent transport of riboflavin, whereas EV membrane vesicles demonstrated minimal transport, which was <20% of the total transport in BCRP membrane vesicles. As can be seen, riboflavin transport was linear for 5 min. Therefore, all the following BCRP-mediated experiments were carried out for 5 min.  Figure 1(b) shows that ATP-dependent riboflavin transport occurs in an osmotically sensitive space (using sucrose to modify osmolarity), with negligible binding.

### 3.1. Effect of 4-Hydroxynonenal (4HNE)

**Figure 2:**
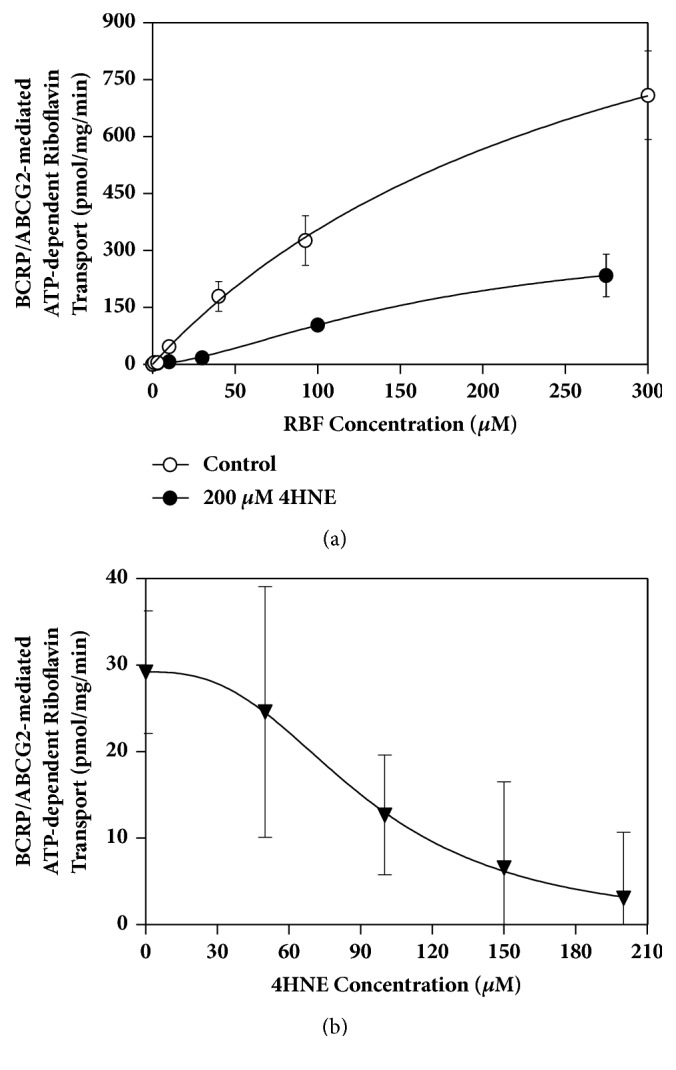
Effect of 4-hydroxynonenal (4HNE) on the transport of riboflavin into* Sf9*-BCRP/ABCG2 membrane vesicles, expressed as pmol riboflavin/mg protein/min. Data are mean ± SD from 3-4 determinations. (a) BCRP-mediated transport of riboflavin in the presence of 200 *μ*M of 4HNE (filled circles) vs. control (open symbols); (b) inhibition of riboflavin uptake by 4-hydroxynonenal (4HNE).

The kinetic parameters for BCRP-mediated transport of riboflavin were assessed by fitting the Hill equation to the data from Figure 2(a). Due to the limited solubility of riboflavin, complete saturation of ABCG2 was not possible to achieve. The maximum concentration of riboflavin tested was 300 *μ*M. The estimates of K_m_ and V_max_ were 296 ± 71 *μ*M and 1407 ± 193 pmol/mg protein/min, respectively, with Hill slope of 1.0. The results of riboflavin uptake in the presence of 200 *μ*M of 4HNE, a product of lipid peroxidation, are also shown in Figure 2(a). The kinetic parameters estimated with Hill equation are 168 ± 32 *μ*M and 342 ± 43 pmol/min/mg for K_m_ and V_max_, respectively, with a Hill coefficient of 1.6 (suggesting modest positive cooperativity). Thus, V_max_ was reduced 4-fold, whereas K_m_ remained unchanged (unpaired* t*-test,* p* > 0.05). These parameters indicate that 4HNE is capable of inactivating BCRP/ABCG2 transport activity. The inhibitory effect of 4HNE (0 to 200 *μ*M) on riboflavin uptake is illustrated in Figure 2(b). The maximal inhibitory concentration (IC_50_) was estimated to be 92 ± 1.4 *μ*M.

### 3.2. Effect of Peroxynitrite (PN)

**Figure 3:**
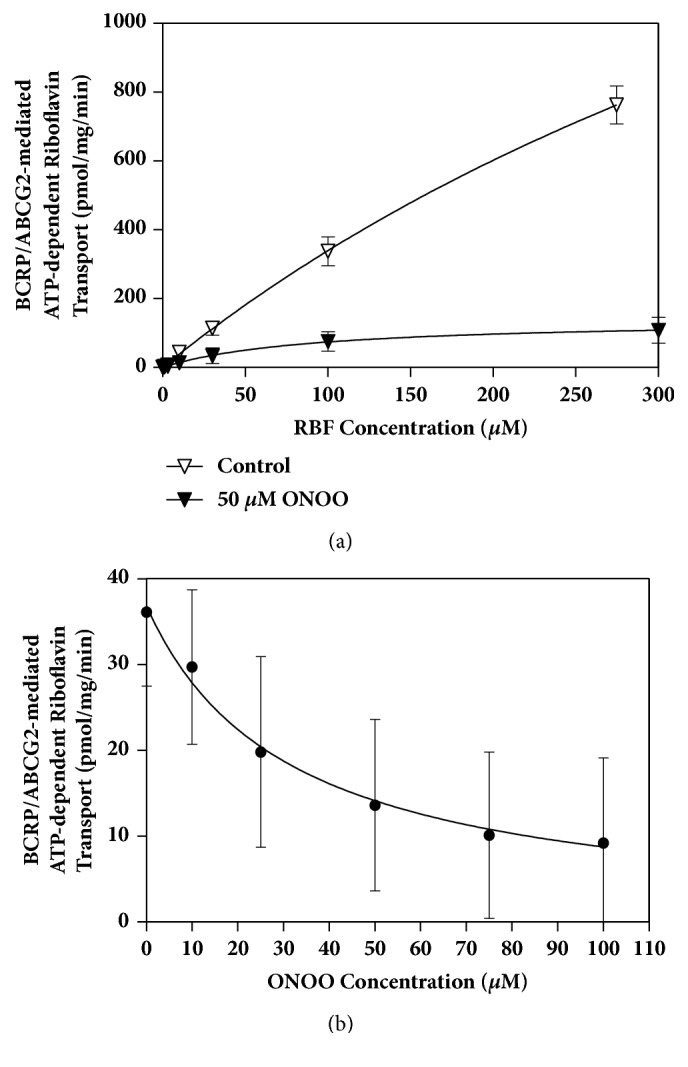
Effect of peroxynitrite (PN) on uptake of riboflavin into* Sf9*-BCRP/ABCG2 membrane vesicles, expressed as pmol riboflavin/mg protein/min. Data are mean ± SD from 3-4 determinations. (a) BCRP-mediated transport of riboflavin in the presence of 50 *μ*M of PN (filled symbols) vs. control (open symbols); (b) inhibition of riboflavin uptake by peroxynitrite (PN).

The kinetic parameters for ATP-dependent riboflavin transport in the presence of an initial concentration of 50 *μ*M of PN were determined by fitting with the Hill equation. Because PN anion is highly unstable at physiological pH, it is supplied in strongly alkaline solutions. Therefore to maintain the reaction buffer at the desired pH of 7.4, a buffer containing triethanolamine HCl (pKa ~ 7.6) instead of tris-HCl was utilized. Because of the changes in buffer constituent, additional experiments were carried out without PN to account for the effects of sodium hydroxide and triethanolamine. The kinetic parameters from these experiments were then compared to those obtained in the presence of 50 *μ*M of PN. The results are shown in Figure 3(a). In the absence of PN, K_m_ and V_max_ were estimated to be 685 ± 167.2 *μ*M and 2663 ± 485.9 pmol/min/mg, respectively, with Hill slope of 1.0. This compares to K_m_ of 90 ± 39.2 *μ*M and V_max_ of 141 ± 23.4 pmol/min/mg, obtained with 50 *μ*M PN, with Hill slope of 1.0. Thus the presence of 50 *μ*M PN drastically reduced V_max_ and Km 19- and 7.5-fold, respectively, suggesting a mixed type of inhibition.

The effect of PN on the riboflavin uptake by* Sf9* membrane vesicles was tested only at initial PN concentrations of up to 100 *μ*M, since at this concentration almost complete inactivation of BCRP was observed. The inhibition curve for BCRP-mediated riboflavin transport is shown in Figure 3(b). IC_50_ was estimated to be 31 ± 2.7 *μ*M indicating that PN is a slightly more potent inhibitor of BCRP/ABCG2 than 4HNE.

## 4. Discussion

It has been demonstrated earlier that riboflavin is a substrate of BCRP [21, 22] and its accumulation in membrane vesicles can be easily measured due to its native fluorescence. In this assay, we made use of this property to rapidly determine BCRP transport activity in a 96-well format with quantitation using a fluorescence plate reader. Previously, a high-speed transport activity assay for BCRP was reported using a 96-well format, with ^3^H-methotrexate as a substrate [23]. However, the present assay was more rapid and less expensive and has less environmental impact than one using radioactivity.

BCRP is expressed at the apical surface of the placental syncytiotrophoblast, where it may be exposed to peroxynitrite and 4-hydroxynonenal released from neutrophils through physiologic and pathophysiologic mechanisms. In the maternal blood circulating through the placenta, activated neutrophils release superoxide as well as lipid peroxides [24]. Superoxide combines with nitric oxide, which is present as an important vasodilator, to form peroxynitrite. Peroxynitrite is highly reactive, particularly forming nitrotyrosine adducts on nearby proteins. Also, lipid peroxidation initiated by the activated neutrophils will result in the formation of reactive aldehydes such as 4HNE and malondialdehyde [25].

4HNE has three main functional groups: an aldehyde, a carbon-carbon double bond, and a hydroxyl group, which can react alone or in sequence of reactions [10]. Due to its lipophilic nature 4HNE has a tendency to accumulate within the lipid bilayer at the site of lipid peroxidation and reach concentrations up to 4.5 mM [26]. Thus, the IC_50_ we reported (92*μ*M) could be observed* in vivo*. However, at concentrations ranging from 1 to 20 *μ*M, 4HNE can already inhibit DNA and protein synthesis, and cytotoxicity is observed at ≥ 100 *μ*M [10]. The inhibition of BCRP/ABCG2 activity occurs slightly slower and concurrently with inhibition of other vital cell processes. One must note however that in the present experiments 4HNE was added to the aqueous buffer. This would require some time for 4HNE to diffuse into the bilayer, whereas* in vivo* 4HNE is usually generated within lipid bilayers. Thus, the IC_50_ values obtained in this study could be overestimated due to possible reactions of 4HNE with other biomolecules before reaching a target transporter [26]. The increase in the Hill coefficient due to 4HNE is not readily explained and would require further studies but could be due to a denaturation of the transporter protein or its lipid environment [27]. Notably, the kinetic parameters in the control experiments differed significantly. This may be due to the change in buffer from tris-HCl to triethanolamine-HCl; furthermore, it may be a result of the 30-minute preincubation step prior to the transport experiments.

Although peroxynitrite anion is relatively short-lived, it not only causes lipid peroxidation but also diffuses inside the lipid bilayer and oxidizes or nitrates integral membrane proteins. Under physiological conditions, PN concentrations can be as high as 50 to 100 *μ*M, but its steady-state concentration is usually in nanomolar range, which can be sustained for a long period of time. [11] Thus, a tissue can have a very high cumulative exposure to PN. Grover et al. [28], for instance, have reported that Ca^2+^ pumps (SERCA) can be inhibited by 50% at PN concentrations of about 50 *μ*M (*in vitro*). Hence IC_50_ of PN obtained in this study (31*μ*M) can be easily observed under physiological conditions, with inactivation of BCRP.

## 5. Conclusion

In conclusion, the present study was conducted to investigate the effect and potency of peroxynitrite (PN) and 4-hydroxynonenal (4HNE), a product of lipid peroxidation, on the activity of BCRP/ABCG2. This investigation revealed that PN (IC_50_ = 31 *μ*M) and 4HNE (IC_50_ = 92 *μ*M) inactivate BCRP/ABCG2 at biologically relevant concentrations. Since the formation of excess PN and 4HNE often takes place during different stages of various disease conditions, PN and 4HNE are capable of decreasing the activity of ABC transporters, which may affect drug disposition.

## Data Availability

The data used to support the findings of this study are available from the corresponding author upon request.

## Abbreviations

4HNE: 4-Hydroxynonenal
ABC: ATP-binding cassette
BCRP: Breast cancer resistance protein/ABCG2
OSP: Oxidative stress products
PN: Peroxynitrite.
